# Waggawagga-CLI: A command-line tool for predicting stable single α-helices (SAH-domains), and the SAH-domain distribution across eukaryotes

**DOI:** 10.1371/journal.pone.0191924

**Published:** 2018-02-14

**Authors:** Dominic Simm, Martin Kollmar

**Affiliations:** 1 Group Systems Biology of Motor Proteins, Department of NMR-based Structural Biology, Max-Planck-Institute for Biophysical Chemistry, Göttingen, Germany; 2 Theoretical Computer Science and Algorithmic Methods, Institute of Computer Science, Georg-August-University Göttingen, Göttingen, Germany; UMR-S1134, INSERM, Université Paris Diderot, INTS, FRANCE

## Abstract

Stable single-alpha helices (SAH-domains) function as rigid connectors and constant force springs between structural domains, and can provide contact surfaces for protein-protein and protein-RNA interactions. SAH-domains mainly consist of charged amino acids and are monomeric and stable in polar solutions, characteristics which distinguish them from coiled-coil domains and intrinsically disordered regions. Although the number of reported SAH-domains is steadily increasing, genome-wide analyses of SAH-domains in eukaryotic genomes are still missing. Here, we present Waggawagga-CLI, a command-line tool for predicting and analysing SAH-domains in protein sequence datasets. Using Waggawagga-CLI we predicted SAH-domains in 24 datasets from eukaryotes across the tree of life. SAH-domains were predicted in 0.5 to 3.5% of the protein-coding content per species. SAH-domains are particularly present in longer proteins supporting their function as structural building block in multi-domain proteins. In human, SAH-domains are mainly used as alternative building blocks not being present in all transcripts of a gene. Gene ontology analysis showed that yeast proteins with SAH-domains are particular enriched in macromolecular complex subunit organization, cellular component biogenesis and RNA metabolic processes, and that they have a strong nuclear and ribonucleoprotein complex localization and function in ribosome and nucleic acid binding. Human proteins with SAH-domains have roles in all types of RNA processing and cytoskeleton organization, and are predicted to function in RNA binding, protein binding involved in cell and cell-cell adhesion, and cytoskeletal protein binding. Waggawagga-CLI allows the user to adjust the stabilizing and destabilizing contribution of amino acid interactions in *i*,*i+3* and *i*,*i+4* spacings, and provides extensive flexibility for user-designed analyses.

## Introduction

Stable single α-helices (SAHs) are extended helices that are not buried within globular structures or coiled-coil helical dimers [1–8]. Their most common function is to serve as rigid connectors or constant force springs between structural domains [1–5,7,9,10], but they also provide contact surfaces for protein-protein and protein-RNA interactions [7,8]. The latter function has been found for the inner centromere protein INCENP and for many regions of spliceosomal proteins in various complexes formed during the pre-mRNA splicing cycle. Independent of any tertiary interactions, SAH-domains are stable and monomeric in polar solvents. These features distinguish SAH-domains from other proteins that fold into α-helices only in the presence of binding partners such as stathmin which is an intrinsically disordered protein lacking any stable fold in the absence of binding partners, but forms an extended α-helix when binding to tubulin dimers [11,12].

SAH-domains are extremely rich in glutamate (E), lysine (K) and arginine (R) [4,6,13,14], which have been shown to stabilize poly-alanine peptides by charge interactions along the helix [15–20]. Although aspartate (D) can also form stabilizing interactions with K/R [21,22], aspartates occur less often than isoleucine, leucine, methionine, alanine and glutamine in predicted, highly likely SAH-domains [13,14]. Especially repeated patterns of four E followed by four K/R seem to stabilize α-helices, while peptides with repeats of two residues do not show helical content [3,17,23]. The specific (E_4_(R/K)_4_)_n_ pattern has therefore been termed *ER/K motif* [6] but this is sometimes mixed up with the term *EK/R α-helix* (e.g. [8]), which had been introduced as alternative term to SAH [3]. Another term introduced in the field is *charged single α-helix* (CSAH) [4] but SAH-domains must not have an overall net charge. Protein regions with stable single α-helices must also not be uninterrupted helices but might include short breaks leading to multiple successive SAHs behaving as a worm-like chain [10]. To exclude misunderstandings because of term usage and to include all special cases, we will refer to these protein regions as SAH-domains from now on.

The experimental identification of SAH-domains first in caldesmon and then L9 ribosomal protein and class-10 myosin has fostered the idea that SAH-domains might be common structural motifs and be present in many other proteins. In first analyses with BLAST using the EK/R motif [3] and the SAH-domain of class-10 myosin [13], 123 distinct proteins in 137 archaea and eukaryotes and 36 human proteins, respectively, have been identified. In a more exhaustive search against UniProt using two newly developed software tools and requiring a minimum of 40 amino acids for an SAH-domain to be detected, SAH-domains were identified in all three kingdoms of life and it was estimated that their abundance is less than 0.2% of all proteins of a species [24]. This, however, was a very conservative approach and less stringent criteria might inevitably yield more SAH-domains. Interestingly, the most SAH-domains were found in the human proteome (165 proteins).

Waggawagga was developed as a web application to visually compare coiled-coil predictions from various tools using helical-wheel and helical-net representations [25]. These representations also allow distinguishing between predicted coiled-coils and SAH-domains. A score summarizing stabilizing and destabilizing interactions was introduced to discriminate SAH-domains from non-SAH-domains. Although most SAH-domains and non-SAH-domains can clearly be discriminated, a large-scale analysis of more than 7900 myosin sequences across all eukaryotes revealed a twilight-zone between the two extremes [14]. Sequences with SAH-domain-scores within this twilight-zone likely need experimental confirmation to demonstrate their SAH or non-SAH appearance. Here, we present a new version of Waggawagga, termed Waggawagga-CLI, intended for the command-line usage to investigate small- and large-scale protein sequence data.

## Results and discussion

Waggawagga-CLI is the command-line version of the web application Waggawagga. It was developed to provide a tool for large-scale SAH-domain prediction and analysis and should, in principle, be able to manage protein sequence datasets of any size. Waggawagga-CLI has not been optimized for speed, but an SAH-domain prediction is likely to be performed only once for each dataset. The SAH-domain predictions are stored in a mobile database thus allowing repeated analyses in case the default parameters need to be adjusted. In contrast to the web application which predicts SAH-domains based on the heptad-repeat assignments of coiled-coil prediction tools, Waggawagga-CLI assumes each protein sequence to be a continuous α-helix and predicts SAH-domains in these.

The Waggawagga-CLI version contains all required secondary software libraries and a lightweight database, SQLite, and therefore does not require any further software installations by the user. The SQLite database is used for storing analysed sequence data during runtime, and can be queried by the advanced user afterwards in multiple ways. Waggawagga-CLI predicts SAH-domains for each of the sequences in the file and subsequently filters the hits by two cut-offs, the minimum SAH-score for each amino acid to be included in an SAH-domain (default: 0.25) and the minimum SAH-domain-score, which depends on the length of the SAH-domain window (default windows: 14, 21, 28, and 49 amino acids). Accordingly, the window size sets the minimum length of an SAH-domain to be detected. The default SAH-domain-scores are based on the results of a comprehensive analysis of more than 7900 myosin sequences across all eukaryotes [14] and range from 0.25 (window 49 aa) to 0.35 (window 14 aa). The results of a Waggawagga-CLI run are provided in text format (and optional gnuplot SVG images) for each sequence containing a predicted SAH-domain, and in summary tables, for each SAH-domain window separately. The summary tables comprise an SAH amino acid distribution analysis, a list of the predicted SAHs by length of SAH-domains, and a detailed list of all SAH-domains with amino acid sequences and SAH-scores for each amino acid.

To demonstrate the application of Waggawagga-CLI on large-scale datasets such as protein sequence datasets generated by whole-genome annotations, we selected protein annotation datasets from species across the eukaryotic tree of life (Table 1). The datasets were obtained from Ensembl Genomes release 87 [26]. The overall runtime per dataset ranged from a few hours to seven days depending on dataset size. The average runtime for single sequences ranged from 4.6 to 23.3 seconds. Across all datasets, the average runtime is 8.3 seconds per sequence which corresponds to 252 aa per second.

**Table 1:** Number of sequences and runtime per protein dataset. All datasets were downloaded from Ensembl Genomes. According to the specifications at Ensembl, datasets specified by “all” represent the super-set of all translations resulting from Ensembl known or novel gene predictions, while datasets specified by “ab initio” include translations resulting from *ab initio* gene prediction software tools. Such *ab initio* predictions are based solely on the genomic sequence and not any other experimental evidence, and, therefore, not all predictions represent biologically real proteins. The sequences listed as *Seqs valid* represent the part of all sequences which meet the necessary conditions for processing (e.g. minimum length of sequence).

Organism	Seqs total	Seqs valid	# AA	CPU hrs.	Time/Seq [sec]
*Arabidopsis thaliana* [ab initio]	20579	20517	42948060	46,5	8,16
*Arabidopsis thaliana* [all]	48321	47952	83278520	82,3	6,17
*Caenorhabditis elegans*	31574	31191	58037112	60,0	6,93
*Chlamydomonas reinhardtii*	14489	14473	26291352	25,9	6,43
*Cyanidioschyzon merolae*	4998	4963	10046308	9,7	7,00
*Danio rerio* [ab initio]	36087	35675	71517964	75,7	7,64
*Danio rerio* [all]	45336	44759	91282168	93,6	7,53
*Dictyostelium discoideum*	13267	13025	28052304	29,4	8,13
*Drosophila melanogaster* [ab initio]	36155	36056	66438088	75,2	7,50
*Drosophila melanogaster* [all]	30362	30194	80128800	110,1	13,13
*Gallus gallus* [ab initio]	50996	49017	85316640	88,5	6,50
*Gallus gallus* [all]	30252	30196	61283944	69,6	8,30
*Giardia lamblia*	7364	6730	12906580	43,5	23,28
*Homo sapiens* [ab initio]	50890	50200	84319768	92,8	6,66
*Homo sapiens* [all]	102915	97110	153405460	181,7	6,74
*Leishmania major*	8308	8307	20934752	21,2	9,18
*Mus musculus* [ab initio]	57111	56142	82405740	86,2	5,53
*Mus musculus* [all]	61440	59075	105206872	118,8	7,24
*Oryza sativa* [ab initio]	63510	63219	124444320	126,5	7,20
*Oryza sativa* [all]	42132	41596	55154072	52,8	4,57
*Plasmodium falciparum*	5352	5350	16345344	17,3	11,67
*Saccharomyces cerevisiae*	6692	6573	12023708	11,6	6,34
*Schizosaccharomyces pombe*	5146	5126	9548000	9,2	6,49
*Tetrahymena thermophila*	24725	24266	64078484	67,8	10,06

### SAH-domain distribution in eukaryotes

**Fig 1:**
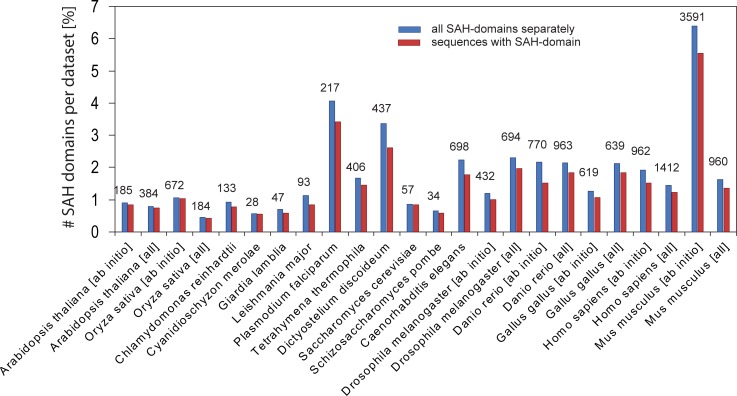
SAH-domains in eukaryotic genomes. The plot presents the percentage of SAH-domains with respect to total numbers of processed sequences per datasets. The blue bars represent all SAH-domains, while the red bars represent all sequences containing at least a single SAH-domain. The numbers on top of the blue bars denote the total numbers of predicted SAHs (see also Table 2). The window size for identifying SAHs was 21 amino acids. For an explanation of the difference between “all” and “ab initio” datasets see Table 1.

SAH-domain validation depends on the length of the sequence window for supposed SAH-domains [14]. Short SAH-domains are not (or considerably less) detected using a large window (e.g. 49 amino acids), and long but less characteristic SAH-domains are often below the cut-off when using a short window (e.g. 14 amino acids). Therefore, by default Waggawagga-CLI determines SAH-domains with four windows, 14, 21, 28, and 49 amino acids. The numbers of detected SAH-domains in the analysed eukaryotic genomes are summarized in Table 2. The lowest total numbers of SAH-domains were found in *Cyanidioschyzon merolae* (28 SAH-domains, 21 aa window), and *Schizosaccharomyces pombe* (34 SAH-domains, 21 aa window), but these species also have the lowest gene numbers (Table 1). In contrast, *Plasmodium falciparum* with only a slightly higher number of genes contains six times more SAH-domains (Table 2). With respect to the percentage of SAH-domains found per dataset, and the percentage of sequences containing SAH-domains per dataset, these species represent the lower (0.5%) and upper (3.5%) limits of the range of SAH-domains per species (Fig 1). The total number of SAH-domains and the number of sequences with SAH-domains are very similar for each dataset indicating that most sequences contain a single SAH-domain. While the datasets of the unicellular species contain one transcript per gene, the *ab initio* and *all* datasets also contain alternative transcripts. These transcripts contain identical, overlapping and independent SAH-domains (see section below). As a rough estimate, the total number of unique SAH-domains per genome is the total number of SAH-domains per datasets divided by the average number of transcripts per gene. In general, we identified less SAH-domains in the *ab initio* datasets than in the *all* datasets (Table 2), except for *Oryza sativa* and *Mus musculus*, where the total numbers of SAH-domains in the *all* and *ab initio* datasets are likely strongly under- and overestimated, respectively (Fig 1). Compared to the only other available genome-wide analysis of SAH-domains [24] we find two to six times more SAH-domains per genome. Given that about 1.5% of all genes of a species contain an SAH-domain, the SAH-domain is not a rare but a widely distributed and used building block for proteins.

**Table 2:** Number of predicted SAH-domains per protein dataset. SAH-domains were filtered by score for windows of 14, 21, 28, and 49 amino acids. Higher numbers for SAH-domains per dataset than sequences with SAH-domains per dataset indicate that some sequences contain multiple independent SAH-domains.

	14		21		28		49	
Organism	#SAH	#Seq	#SAH	#Seq	#SAH	#Seq	#SAH	#Seq
*Arabidopsis thaliana* [ab initio]	151	138	185	173	132	123	86	81
*Arabidopsis thaliana* [all]	306	270	384	352	272	244	181	165
*Caenorhabditis elegans*	633	506	698	558	562	460	407	325
*Chlamydomonas reinhardtii*	119	100	133	113	95	83	63	55
*Cyanidioschyzon merolae*	22	21	28	27	9	9	5	5
*Danio rerio* [ab initio]	751	479	770	544	537	392	377	279
*Danio rerio* [all]	877	747	963	821	687	604	474	415
*Dictyostelium discoideum*	408	318	437	339	331	273	227	197
*Drosophila melanogaster* [ab initio]	383	330	432	360	289	249	168	148
*Drosophila melanogaster* [all]	620	546	694	592	456	398	302	266
*Gallus gallus* [ab initio]	546	462	619	515	456	391	305	280
*Gallus gallus* [all]	549	482	639	557	435	383	263	243
*Giardia lamblia*	36	30	47	39	27	23	17	16
*Homo sapiens* [ab initio]	887	694	962	767	663	562	441	379
*Homo sapiens* [all]	1262	1044	1412	1181	969	829	621	539
*Leishmania major*	70	57	93	71	56	41	42	29
*Mus musculus* [ab initio]	3403	2931	3591	3108	2780	2522	1847	1722
*Mus musculus* [all]	844	734	960	804	655	589	450	399
*Oryza sativa* [ab initio]	487	475	672	655	423	417	387	383
*Oryza sativa* [all]	161	156	184	180	126	123	77	75
*Plasmodium falciparum*	167	142	217	183	148	130	130	112
*Saccharomyces cerevisiae*	49	46	57	55	34	34	21	21
*Schizosaccharomyces pombe*	31	26	34	30	19	17	11	11
*Tetrahymena thermophila*	352	306	406	350	266	240	174	158

**Fig 2:**
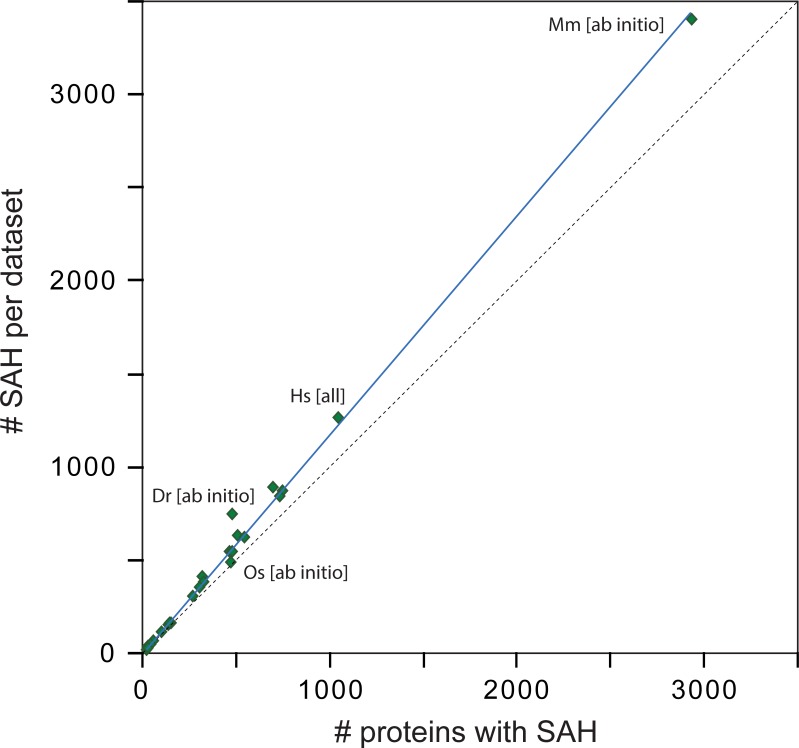
SAH-domains in eukaryotic genomes. The plot contrasts the total number of SAH-domains per dataset with the total number of sequences containing at least a single SAH-domain. Each diamond represents a dataset. The number of sequences with multiple SAH-domains is not a characteristic of certain species but depends on the total number of sequences with SAH-domains per species. The more sequences contain SAH-domains, the more sequences with multiple SAH-domains will be found. For orientation, labels were given for likely the best annotated dataset, human [all], the most extreme case mouse [ab initio], and the two datasets with the largest deviation from the line, danio [ab initio] and rice [ab initio]. The datasets with SAH-domains identified with a window size of 14 amino acids were taken. Abbreviations: Dr, *Danio rerio*; Hs, *Homo sapiens*; Mm, *Mus musculus*; Os, *Oryza sativa*.

The number of protein sequences with multiple SAH-domains linearly depends on the total number of sequences with SAH-domains (Fig 2). Thus, species-independent about 15% of the sequences with an SAH-domain contain at least one further SAH-domain. Only flowering plants, *Cyanidioschyzon merolae*, and *Saccharomyces cerevisiae* have considerably less genes with multiple SAH-domains.

### SAH-domains in human alternative transcripts and evolution

**Fig 3:**
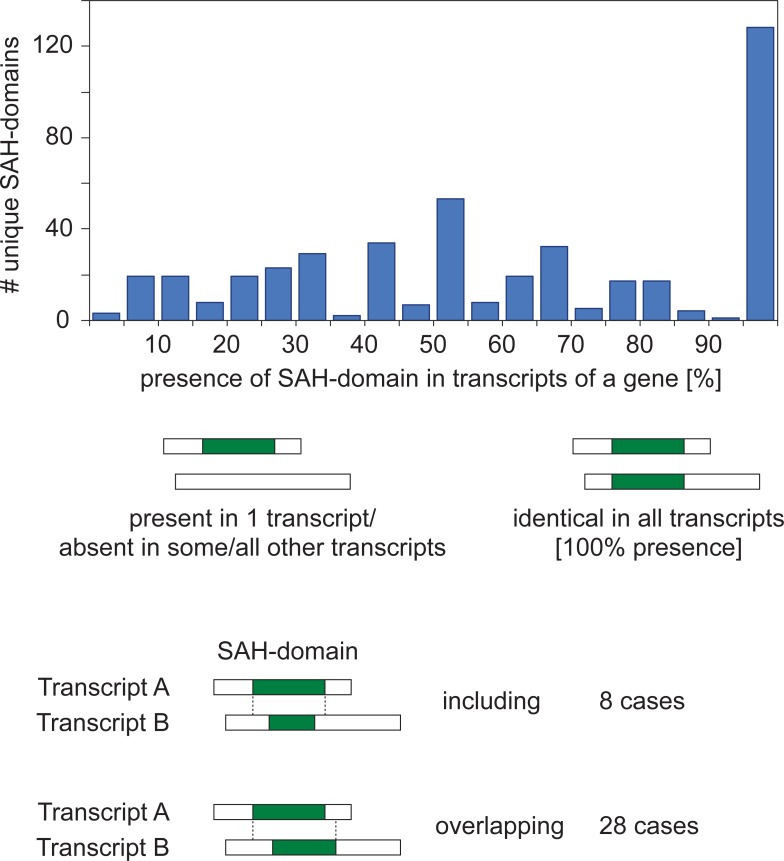
Presence of SAH-domains in alternative human transcripts. All predicted SAH-domains from the human “all” dataset were compared across transcripts from the same gene and combined to *unique* SAH-domains in case of identical sequences. For each unique SAH-domain its presence or absence in all annotated transcripts of the gene was determined. The histogram represents the fraction of unique SAH-domains present in alternative transcripts, with each bin combining values of a range of 5%. The dataset also contains a few cases where a unique SAH-domain is completely part of a larger unique SAH-domain of another transcript and where unique SAH-domains from different transcripts overlap.

SAH-domains are structural entities in proteins, and it seems likely that extended, combined, and altered SAH-domains might be obtained as result of alternative splicing. More simply, SAH-domains might either be present or absent in protein variants of a gene. A previous analysis of SAH-domains across human and mouse transcripts identified nine and seven cases, respectively, where the SAH-domains are either present or absent in alternative transcripts [24]. For a single human gene, *AFDN* (protein: afadin), transcripts resulting in SAH-domains of different length were found. Because the human genome annotation is likely the most complete comprising extensive alternative transcripts we used the human dataset “all” to analyse the role of SAH-domains as structural building block. We distinguish three cases of SAH-domains resulting from a single gene (presence/absence, including, overlapping; Fig 3), and all can happen at the same time if a gene is spliced in more than two alternative transcripts, or is spliced in two alternative transcripts and encodes multiple SAH-domains.

The human dataset “all” contains 97110 transcripts (Table 1) which are derived from 22622 genes. We identified 1262 SAH-domains (14 aa window, Table 2), of which 447 are unique with respect to genes. Of the 22622 genes, 5577 code for a single and 17045 code for multiple transcripts. 31 (0.55%) of the single and 265 (1.55%) of the multiple transcript genes encode 51 and 396 unique SAH-domains, respectively. 77 unique SAH-domains are present in all transcripts of the multiple transcript genes while 319 (80.6%) unique SAH-domains are absent in at least one of the transcripts (Fig 3). 116 unique SAH-domains are present in only a single from up to 24 transcripts. We identified only eight cases where unique SAH-domains are completely part of larger SAH-domains in other transcripts (case *including*). 28 unique SAH-domains overlap with unique SAH-domains of other transcripts of the same gene (case *overlapping*; minimum number of overlapping amino acids: 5).

The presence of an SAH-domain region in all transcripts of a gene indicates that the respective region is an essential structural entity in all resulting proteins. Our analysis shows that SAH-domains are indispensable in transcripts of only 19.4% of the genes. In the majority of the cases, SAH-domains are differentially included building blocks and add to the diversity of protein isoforms. Modulation of the SAH-domain lengths (cases *including* and *overlapping*) happens but is currently rare.

If SAH-domains represent structural building blocks similar to any other structural domain they should appear in transcripts of orthologous and paralogous proteins. If SAH-domains are part of exon shuffling processes they might appear in unrelated proteins. Because of the low amino acid and structural complexity in SAH-domains few mutations could turn these motifs into intrinsically disordered regions (which still might fold into α-helices upon interaction with binding partners) or α-helices that aggregate or even form coiled-coil structures. Thus, sequence homology based methods likely do not provide any specific relations. Instead, we searched for identical sequences of unique SAH-domains across all genes. Of the 447 human SAH-domains, 383 are unique with respect to the human genome. 30 SAH-domains are present in identical sequence in two to seven different genes. Most of these belong to gene paralogs such as multiple *golgin A6* family and *golgin A8* family genes, *tropomyosin 3* and *tropomyosin 4*, and the calcium channel subunits *CACNA1H* and *CACNA1I*. In addition to these identical SAH-domains we identified 68 cases where a unique SAH-domain from one protein is part of a longer SAH-domain in another protein (case *including*).

### Characteristics of SAH-domains

**Fig 4:**
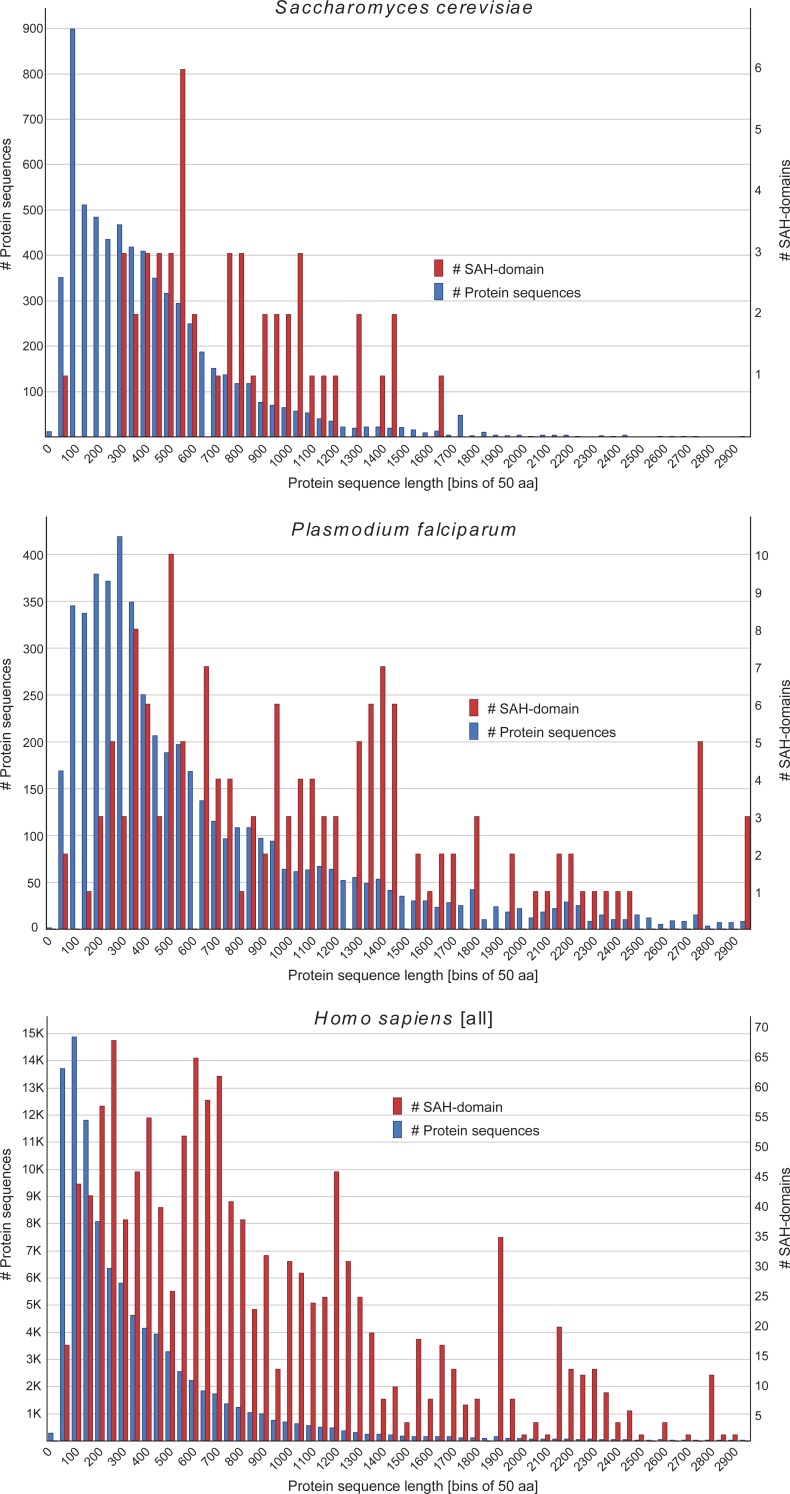
Distribution of SAH-domains with respect to protein sequence length. As examples for comparing the lengths of protein sequences in general with the presence of SAH-domains in proteins, the proteins from the *Plasmodium falciparum*, *Saccharomyces cerevisiae* and human [all] datasets were plotted. The latter proteins comprise the translations of all annotated gene transcripts. Proteins were combined in bins of 50 amino acids for better visualization, and proteins with length >3000 aa were omitted. All SAH-domains identified with the 14 aa window are counted and shown.

SAH-domains are regions with low sequence homology and low amino acid diversity, and their comparison is therefore restricted to some basic metrics. In all species analysed SAH-domains are enriched in longer proteins (Fig 4). This is consistent with their main function as connectors between other protein domains, although they might also function as direct binding site for other proteins. Because of their limited structural and amino acid diversity, direct protein-protein and protein-RNA binding via the SAH-domains is likely very rare. Mostly, SAH-domains are anchored to additional domains such as those in myosins [2,5], the Inner Centromere Protein (INCENP) [7], and in the spliceosomal proteins MFAP1 and Snu23 [8]. Because of this combination with other domains, which has also been found in an earlier analysis [24], it is evident that SAH-domains are rather found in longer protein sequences.

Another characteristic of SAH-domains distinguishing them from any other domain is their amino acid distribution with up to 80% of the residues being E, K and R [4,13,24]. Of the various possibilities to build salt bridges, E→R was shown in short peptides to be more α-helix stabilizing than E→K [27], and both are more stabilizing than the reverse salt bridges R→E and K→E. E→R salt bridges are also the most favourable for the speed of folding [22]. However, comparison of long repeats of AEEEXXX with X being either K or R showed, that such peptides aggregated when two or three of the X were R [28]. A repeat including one arginine, however, was more helical and stable than a repeat with only lysines. Repeats with three or five alanines per heptad also aggregated [28]. Thus, it seems that a certain percentage of charged amino acids and a mixture of arginines and lysines are the most favourable to stabilize single α-helices.

**Fig 5:**
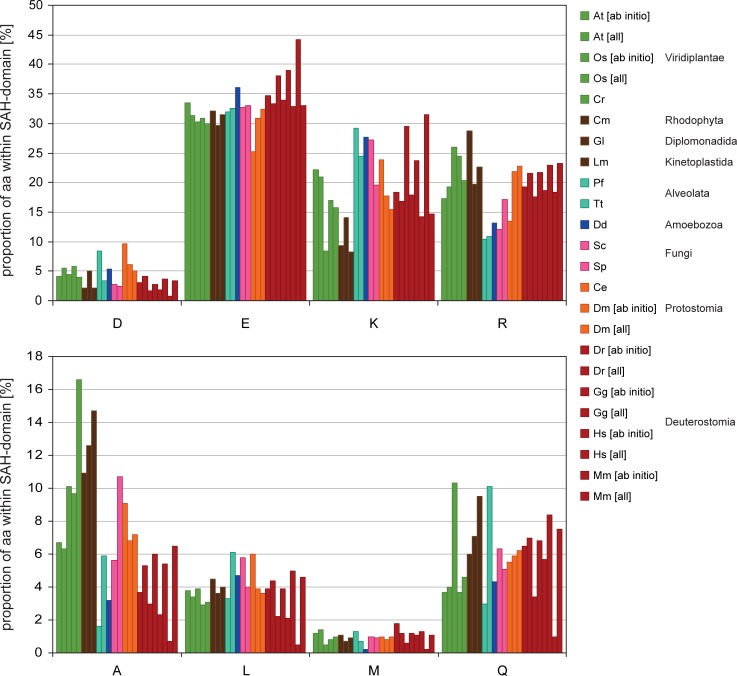
Proportion of the most abundant amino acids within SAH-domains with respect to dataset and eukaryotic domain. For computing the amino acid proportions, the SAH-domains predicted with the 14 aa window were used. The proportions within the four windows are almost identical for each species.

**Fig 6:**
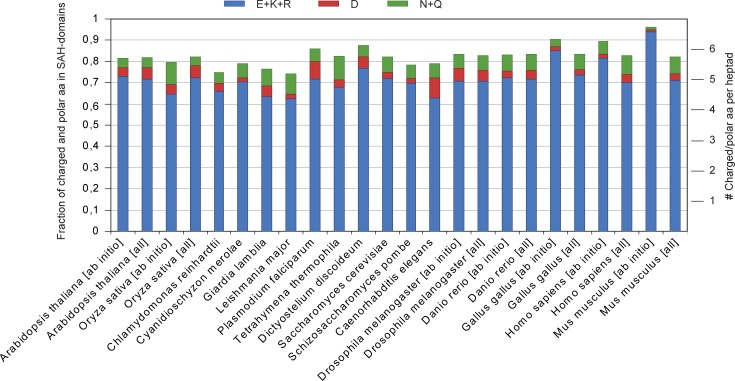
Fraction of charged and polar amino acids within SAH-domains across species. For better orientation, fractions are also given for distinct numbers of heptad positions fully occupied by charged/polar amino acids.

The analysis of the amino acid distribution in the predicted SAH-domains across the eukaryotes shows that aspartate is rarely used in SAH-domains and that glutamate is the dominating negatively charged amino acid (Fig 5). This is consistent with earlier extrapolations from few data [4,13,24] and in agreement with studies on short peptides showing considerably less stabilizing effects of D→K and D→R salt bridges compared to their glutamate homologs [21,23]. Across the eukaryotes there are strong differences for the preference of the positively charged amino acid (Fig 5). In plants, green and red algae, diplomonads, kinetoplastids, and metazoans, arginines are preferred to lysines, while lysines are strongly preferred in alveolates, amoebae, and yeasts. The finding of twice as many arginines than lysines in red algae, diplomonads, and kinetoplastids seems to contract the findings of aggregating peptides with similar arginine to lysine proportions [28]. However, these peptides were based on repeats of three negatively and three positively charged amino acids, and natural peptides might form stabile single α-helices if the same salt bridges were randomly distributed and not present in such ordered three-to-three repeats. In total, about 80% of the supposed heptad positions are occupied by charged residues or asparagine and glutamine, which corresponds to about 5,5 heptad positions (Fig 6). Species with, in total, less E/K/R have more aspartates, asparagines or glutamines. This also corresponds with the results obtained from the AEEEXXX repeats that showed that peptides aggregated if three or more positions of the heptads are occupied by alanines [28]. Alanines show the widest distribution from all uncharged amino acids found to be present in SAH-domains (Fig 5), with the highest fractions found in green and red algae, diplomonads, and kinetoplastids. In contrast, alanines are rarely present in *Plasmodium falciparum* and *Dictyostelium discoideum* SAH-domains.

**Fig 7:**
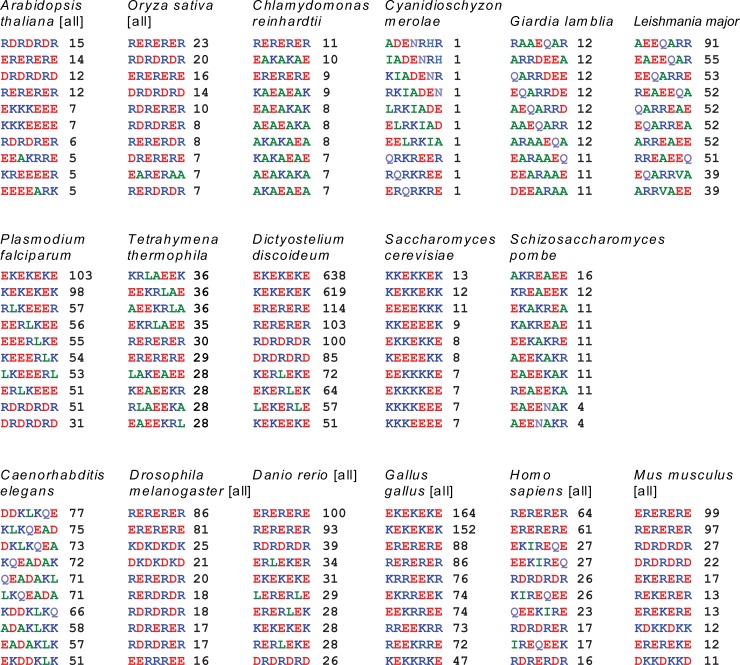
The ten most common heptad repeats found in SAH-domains per species. For each dataset of SAH-domains predicted using the 14 amino acid window all possible heptads were determined and ranked by frequency, given as numbers next to each heptad sequence.

Next we were interested in identifying the most common patterns in natural SAH-domains (Fig 7). Compared to total numbers of SAH-domains and lengths of SAH-domains the most common heptad repeats are rather rare within each species. For example, the most common heptad pattern in human SAH-domains, RERERER, is present in only 3.6% of the sequences with SAH-domains and accounts for only 0.7% of the amino acids of all SAH-domains. This indicates that SAH-domains do not have certain, common patterns but instead are highly variable with respect to their sequences. The most common patterns across most species contain duplets of oppositely charged amino acids, [D/E][K/R]. To our knowledge, such repeats have not extensively been studied experimentally yet. Repeats of (EK)_n_ were shown to be at least partially helical [23], but it is not known whether these are monomeric or aggregate, or even form stable single α-helices at all. However, only a minor fraction of the SAH-domains exclusively consists of such [D/E][K/R] repeats. In most cases, such repeats are part of predicted SAH-domains with variable sequence such as the SAH-domain in human Myo10, “AEKREQEEKKKQEEEEKKKREEEE**RERERER**REAELRAQQEEETRKQQELEALQKSQKEAEL” (ENSP00000421280). We suspect that these [D/E][K/R] repeats are enriched in the analysis because of their very simple pattern. If regions exclusively consisting of [D/E][K/R] repeats do not form stable single α-helices, a small fraction of false-positive predictions might be present in a Waggawagga analysis.

Other common heptad patterns contain alanines and glutamines (Fig 7) and resemble the widely studied peptides based on poly-alanine backbones onto which oppositely charged amino acids were placed in all possible spacings. Heptad patterns based on oppositely charged amino acid triplets such as (AE_3_[K/R]_3_)_n_ [28] were not found. Octad patterns with quadruplets of glutamates and arginines/lysines (E_4_[K/R]_4_), which have systematically been studied experimentally [3,23], are extremely rare in natural SAH-domains of eukaryotes. Also, they are not present in repeated patterns indicating that these octad patterns appeared by chance but are in fact part of heptad patterns. If clusters of identical amino acids were found, then these appear in repeated heptad patterns from triplets and quadruplets (Fig 7, see patterns of *Arabidopsis thaliana* and *Saccharomyces cerevisiae*, for example).

Although heptad patterns are shown in Fig 7, most of these patterns are built from even-numbered smaller motifs. Heptad repeats tend to form supra-molecular structures because the repeat moves around the helix axis, as is evident from coiled-coil structures, where hydrophobic residues form a left-handed helical seam along the surface of the right-handed α-helix which is buried in the centre of the dimer. In contrast, patterns of even-numbered motifs are supposed to form straight α-helices and to rather aggregate in stacks, if at all, than in intertwined supra-molecular helices.

### Functional analysis of SAH-domains in *Saccharomyces cerevisiae* and human

To determine whether proteins with SAH-domains are particularly involved in certain cellular processes, localizations and molecular functions, we performed gene ontology enrichment analyses of the *Saccharomyces cerevisiae* and human datasets. Yeast (*Saccharomyces cerevisiae*) proteins with SAH-domains have not been analysed at a whole-genome level yet, because yeast was not among the species with highest numbers of SAH-domain containing proteins in an UniProt analysis [24]. Yeast proteins with SAH-domains are particularly enriched in the GO terms macromolecular complex subunit organization, cellular component biogenesis and RNA metabolic processes. Enriched GO terms also include nuclear and ribonucleoprotein complex localization, and function in ribosome and nucleic acid binding. Cytoskeleton associated processes and functions were not among the enriched terms for the yeast proteins. The recent discovery of a few spliceosomal proteins with potential SAH-domains [8] is consistent with the observed enrichment of the yeast proteins.

The human proteins with SAH-domains have roles in all types of RNA processing (mRNA processing, mRNA metabolic processes, RNA splicing) and cytoskeleton organization. They are enriched in cytoskeleton and adherens junction localization terms, and are predicted to function in RNA binding, protein binding involved in cell and cell-cell adhesion, and cytoskeletal protein binding. In contrast to earlier analyses based on considerably smaller datasets of human proteins with SAH-domains that showed a wide distribution of cellular localizations and functions [4,13,24], the analysis of our extended whole-genome based dataset demonstrates a strong enrichment in RNA- and cytoskeleton-related processes and localizations.

### Customized usage of Waggawagga-CLI

SAH-predictions are based on scoring statistics. In our previous analysis of almost 8000 myosins from species across all eukaryotes we showed that the scores from SAH-predictions were scoring-window-dependent and could be placed on a continuously increasing line [14]. The exponential shape of the curve allowed defining a lower and an upper cut-off, below which and above which the probability to not-have and to have, respectively, identified an SAH-domain is high. The scores between these cut-offs, in the so-called “twilight”-zone, do not allow a simple “is” or “is not” decision. To provide full flexibility to the prediction and analysis of SAH-domains, Waggawagga-CLI allows adjusting the scoring matrices and the cut-offs for result filtering. There are two amino acid matrix files, one each for amino acids in (*i*, *i+3*) and in (*i*, *i+4*) spacings, where stabilizing and destabilizing interaction scores can be defined for every amino acid combination. Modifying these files allows, for example, fine-tuning the search for specific sub-types of SAH-domains or adapting to species- or lineage-specific parameters such as general amino acid usage. Waggawagga-CLI also allows separating the steps of SAH-domain prediction and analyses so that users experienced in database interaction can easily design additional analyses.

## Conclusions

Waggawagga-CLI is a complementary tool to predict functional domains in whole genome annotations. Our predictions included all previously proposed SAH-domains as far as they were particularly named such as the 36 human proteins identified by BLAST [13]. While BLAST is based on sequence homology, Waggawagga-CLI uses a dedicated scoring scheme for potential *i*,*i+3* and *i*,*i+4* interactions along the sequence and we were, therefore, able to predict more than ten times more SAH-domains. Waggawagga-CLI is thus similar to other SAH-domain prediction software such as SCAN4CSAH and FT_CHARGE [24] but, in contrast to these, allows users to modify the scoring schemes and to adjust cut-off parameters to individual analysis needs. Several proteins from spliceosomal complexes have recently been suggested to contain SAH-domains [8] but most of these rather represent isolated α-helical segments that folded upon binding to other proteins. In MFAP1, the regions with considerable SAH-score are shorter than the minimum window of 14 amino acids that we used in our analysis. There were few potential charged interactions along the α-helix of MFAP1 [8] and similar numbers of potential charged interactions are found for most predicted coiled-coil regions. We suppose that by altering Waggawagga’s scoring scheme to include MFAP1 and similar proteins many false positive coiled-coil proteins will also appear in the list. Adjusting the scoring scheme might, however, be useful if subsets of proteins are analysed that likely do not contain any coiled-coil proteins.

## Materials and methods

### Implementation

Waggawagga-CLI is available for the main operating systems Linux (arch. x86 and 64-Bit) and macOS (10.10 or higher) [and Windows]. It comes in precompiled system-specific packages with a portable Ruby environment (Traveling-Ruby) and a mobile SQLite-database, where the analysed sequence data are stored for direct or later use. Waggawagga-CLI runs out of the box, not requiring any further installations after downloading and extracting the tarball.

In general, to be fully functional the software requires Ruby version 1.9 or later, and the following gems: Active-Record (> 4.0.x), BioRuby (1.5.0) [29], and SQLite3 (1.3.9). Besides the textual result files, full-sequence prediction score graphs (depicted along the full sequences) are additionally available as SVG, if the graphical toolkit GnuPlot (http://www.gnuplot.info) is pre-installed on the user’s system.

### Waggawagga-CLI in contrast to webserver Waggawagga

The focus of Waggawagga-CLI is explicitly on the SAH-domain prediction in large protein sequence datasets (instead of just one at a time). The CLI-version analysis workflow separates prediction and analysis. Sequences are parsed, scored and imported in a single step and can be evaluated with different parameter preferences later-on. The scoring process itself can be adjusted as well, by modifying the pre-installed scoring matrices. The application should run with FASTA-files of any size, although it was tested with only the datasets presented here. The prediction result files are strictly organized in subfolders in the results directory named by the working title parameter (‘id’). Only results with scores above the initial cut-off are kept for later inspection by the user.

### SAH scoring algorithm

The SAH prediction is based on the classical *helical net* diagram which is the representation of a single α-helix opened along a line parallel to its axis and laid flat [30]. Each sequence is considered as a continuous right-handed α-helix and each position in the helix is assumed to be repeated every eighth amino acid. Thus, each sequence can be depicted as a repeat of heptads in the *helical net* diagram. The amino acid positions within each heptad are formalized as letters *a-g* and the first amino acid of each sequence is set to position *a* of the first heptad. This is an important difference to the Waggawagga-webinterface version, where the positions of the amino acids within the heptads are derived from the coiled-coil prediction tools and accordingly allow for gaps and any other pattern within the *helical net* diagram. According to the classical representation the α-helix in the *helical net* diagram is opened along the *f* column. In contrast to the classical representation, which is read from bottom to top, we adopted the view introduced for SAH-domains in which SAH sequences are depicted from top to bottom [2,13] and which seems to be used by most if not all in the SAH community.

For each position in the *helical net* amino acid interactions between interacting residues in *i*,*i+3* and *i*,*i+4* distance are drawn. These interactions are classified into strong, medium and weak stabilizing types, a helix-supporting type and types of destabilizing interactions. But this is only a linguistic differentiation; the interactions can be assigned every possible score. In Waggawagga-CLI, the values for possible amino acid interactions are taken from customizable scoring matrices. The standard scoring files are located in the *config*-directory, are named *‘scoring_matrix_i_3*.*csv’* (for interactions in *i*,*i+3* distance) and *‘scoring_matrix_i_4*.*csv’* (for interactions in *i*,*i+4* distance; S1 Fig), and can freely be edited. In addition to these binary amino acid interactions, we consider two types of interaction networks: i) Subsequent hydrophobic amino acids (V, I, L, M, F, Y) in *i*,*i+3*,*i+6*, *i*,*i+3*,*i+7*, *i*,*i+4*,*i+7* and *i*,*i+4*,*i+8* distance are regarded as destabilizing (because of potentially stabilizing hydrophobic seams in coiled-coil dimers) and contribute a negative network-score. ii) Subsequent oppositely charged amino acids in *i*,*i+3*,*i+6*, *i*,*i+3*,*i+7*, *i*,*i+4*,*i+7* and *i*,*i+4*,*i+8* distance are known to stabilize α-helices more than the sum of the respective binary interactions, and thus contribute an additional positive network-score. These network-scores can be adjusted in the config-file. In contrast to the SCAN4CSH algorithm, we did not consider interactions of identically charged residues in *i*,*i+3* and *i*,*i+4* distance, and of oppositely charged amino acids in *i*,*i+1* and *i*,*i+2* distance, which were regarded as destabilizing [4]. Because such interactions are present in most patterns that are regarded as exemplary SAH-domains, e.g. EEEEKKK, we suspect that inclusion of these interactions does not help in distinguishing between SAH-domains and non-SAH-domains.

### Computing the SAH-score

For computing an SAH-score, all amino acid interaction scores in a *helical net* representation of a certain sequence window are summed up. By default, Waggawagga-CLI computes SAH-scores for windows of 14, 21, 28 or 49 amino acids. Because we consider the protein query sequence to be a continuous α-helix there are also interactions to and from amino acids at the first and the last helical turn within the scoring window. By definition we include all amino acid interactions from residues of the last helical turn of the window (positions *d-g*) to amino acids of the next heptad in the SAH-score. The sum of the interaction scores of each window is then normalized with respect to the highest possible score for the respective window, which is obtained by summing all interactions of an EEEEKKK repeat and which is set to “1”. If individual interaction scores are changed in the scoring matrices (see above), the highest possible scores for each window need to be adjusted accordingly. This is done in the config-file. The user needs to keep in mind that the heptad repeat pattern that will result in the highest possible score, might also change when the scoring matrices change. This interplay between individual interaction scores and accordingly unlimited possibilities for the pattern resulting in the highest possible score is the reason why we keep the window sizes for computing SAH-scores fixed. The SAH-score for the respective window is then assigned to the central amino acid (windows 21 and 49) or amino acids 8 and 15 (windows 14 and 28) of the window, respectively. Scores for the first and last 7, 10, 14 and 24 amino acids (depending on window size) of the protein sequences are calculated by filling the window with dummy amino acids at the N- or C-terminus. By definition, these dummy residues are strictly neutral and do not have interactions with other amino acids. The computed SAH-scores vary considerably from amino acid to amino acid along the sequence depending on how many interactions are lost and gained on each side of the amino acid window. To term a protein sequence region an SAH-domain, the scores of all amino acids within this region need to be above a user-defined SAH-score cut-off (default: 0.25).

### Computing the SAH-domain-score

To be able to compare and rank SAH-domains, we developed the SAH-domain-score. By definition, SAH-domains have a minimum length of 14 amino acids with SAH-scores for each of the 14 amino acids above the SAH-score cut-off. 20% of the amino acids within the full SAH-domain are allowed to have SAH-scores below the cut-off to avoid splitting long SAH-domains into multiple short SAH-domains separated by just one or a few amino acids. However, SAH-domains need to start and to end with amino acids having SAH-scores above the cut-off (default: 0.25). Taking the average of all SAH-scores within an SAH-domain as SAH-domain-score would introduce strong bias by peak values and therefore strongly influence a comparison of short with long SAH-domains. To allow length-independent comparison of SAH-domains we thus developed a score based on a certain amino acid window. Accordingly, the SAH-domain-score is the maximum of the scores computed as the average of SAH-scores within a window of neighbouring amino acids (default: 14 amino acids). For example, given an SAH-domain of 20 amino acids (scores of all 20 aa above the 0.25 score cut-off) six SAH-domain-scores (each possible 14 aa window) are calculated and the highest of these scores is taken as SAH-domain-score for the respective SAH-domain. The length of the window for determining SAH-domain-scores can be adjusted (Advanced Mode parameter).

### Data sources and analyses

The following protein sequence files were downloaded from Ensembl release-87 (ftp://ftp.ensembl.org/pub/release-87/): Arabidopsis_thaliana.TAIR10.pep.abinitio, Arabidopsis_thaliana.TAIR10.pep.all, Caenorhabditis_elegans.WBcel235.pep.all, Chlamydomonas_reinhardtii.v3.1.pep.all, Cyanidioschyzon_merolae.ASM9120v1.pep.all, Danio_rerio.GRCz10.pep.abinitio, Danio_rerio.GRCz10.pep.all, Dictyostelium_discoideum.dicty_2.7.pep.all, Drosophila_melanogaster.BDGP6.pep.abinitio, Drosophila_melanogaster.BDGP6.pep.all, Gallus_gallus.Gallus_gallus-5.0.pep.abinitio, Gallus_gallus.Gallus_gallus-5.0.pep.all, Giardia_lamblia.GCA_000002435.1.pep.all, Homo_sapiens.GRCh38.pep.abinitio, Homo_sapiens.GRCh38.pep.all, Leishmania_major.ASM272v2.pep.all, Mus_musculus.GRCm38.pep.abinitio, Mus_musculus.GRCm38.pep.all, Oryza_sativa.IRGSP-1.0.pep.abinitio, Oryza_sativa.IRGSP-1.0.pep.all, Plasmodium_falciparum.ASM276v1.pep.all, Saccharomyces_cerevisiae.R64-1-1.pep.all, Schizosaccharomyces_pombe.ASM294v2.pep.all, Tetrahymena_thermophila.JCVI-TTA1-2.2.pep.all. The SAH-domain predictions presented and analysed in this study were produced with the latest Waggawagga-CLI version using default parameters.

### Gene Ontology enrichment analysis

Gene Ontology enrichment analyses were done with WebGestalt [31]. The lists of unique genes in gene symbol format were uploaded to WebGestalt and the GO Enrichment Analysis selected. The entire *Saccharomyces cerevisiae* and human genome annotations, respectively, were set as background and 0.05 as threshold for the p-value for the significance test using the default statistical method "hypergeometric”.

### Software and data availability

Waggawagga-CLI is available for download from http://waggawagga.motorprotein.de. The software and all results from data analysis as presented in this study (SAH-domain predictions and GO analyses) are also available from figshare (doi: 10.6084/m9.figshare.5435947).

## Supporting information

S1 Fig Composition of the SAH-score. The figure shows publications and the respective peptides and amino acid interactions analysed. From the reported observed helicities and measured energies of side chain interactions we build the scoring scheme shown in the table at the bottom. The table lists the score of each interaction taken for computing the SAH-score.
(PDF)
