# Supplementary material for: Waggawagga-CLI: A command-line tool for predicting stable single α-helices (SAH-domains), and the SAH-domain distribution across eukaryotes
Source: PLoS One. 2018 Feb 14;13(2):e0191924. doi: 10.1371/journal.pone.0191924 (PMC5812594; doi:10.1371/journal.pone.0191924)
Supplement: S1 Fig — The figure shows publications and the respective peptides and amino acid interactions analysed. From the reported observed helicities and measured energies of side chain interactions we build the scoring scheme shown in the table at the bottom. The table lists the score of each interaction taken for computing the SAH-score. (PDF) [file pone.0191924.s001.pdf]

Marquesee S, Baldwin RL. Helix stabilization by Glu-. . .Lys+ salt bridges in short peptides of de novo design. *Proc Natl Acad Sci*. **1987**; 84: 8898-8902

		-[θ]222
i+4 E,K	A E A A A K E A A A K E A A A K A	29,000
i+4 K,E	A K A A A E K A A A E K A A A E A	25,300
i+3 E,K	A E A A K A E A A K A E A A K A	17,600
i+3 K,E	A K A A E A K A A E A K A A E A	8,500

helix content
(pH 7.0, 1°C, 0.01 M NaCl)

Huyghues-Despointes BM, Scholtz JM, Baldwin RL. Helical peptides with three pairs of Asp-Arg and Glu-Arg residues in different orientations and spacings. *Protein Sci*. **1993**; 2: 80-85

		-[θ]222
i+4 D,R	A D A A A R D A A A R D A A A R Y	25,200
i+4 R,D	A R A A A D R A A A R D A A A R Y	16,300
i+3 D,R	A D A A R A D A A R A D A A R Y	14,000
i+3 R,D	A R A A D A R A A D A R A A D Y	2,200
i+4 E,R	A E A A A R E A A A R E A A A R Y	31,300
i+4 R,E	A R A A A E R A A A E R A A A E Y	22,400
i+3 E,R	A E A A R A E A A R A E A A R Y	22,500
i+3 R,E	A R A A E A R A A E A R A A E Y	11,800

helix content
(pH 7.0, 1°C, 0.01 M NaCl)

Scholtz JM, Qian H, Robbins VH, Baldwin RL. The energetics of ion-pair and hydrogen-bonding interactions in a helical peptide. *Biochemistry*. **1993**; 32: 9668-9676

		ΔG° (E^-K^+)	ΔG° (E^0K^+)
i+4 Q,E	A A Q A A A E Q A A A A Q A A Y	-340	-380
i+4 Q,E	A A Q A A A A Q A A A E Q A A Y	-270	-300
i+1 E,K	A A Q A E K A Q E A A A Q A A Y	200	0
i+2 E,K	A A Q A E A K Q A A E A Q A A Y	180	-95
i+3 E,K	A A Q A A E A Q K A A E Q A A Y	-380	-285
i+4 E,K	A A Q A A E A Q A K A A Q E A Y	-470	-340
i+3 K,E	A A Q A A K A Q E A A E Q A A Y	-460	-320
i+4 K,E	A A Q A A K A Q A E A A Q E A Y	-380	-290

Interactions
(0.01 M NaCl)

Smith JS, Scholtz JM. Energetics of polar side-chain interactions in helical peptides: salt effects on ion pairs and hydrogen bonds. *Biochemistry*. **1998**; 37: 33/40

		-[θ]222	ΔG° (E^-K^+)
i+3 E,K	A A Q A A E A Q K A A A Q A A Y	11,400	-290
i+4 E,K	A A Q A A E A Q A K A A Q A A Y	12,750	-415
i+5 E,K	A A Q A A E A Q A A K A Q A A Y	8,500	
i+3 K,E	A A Q A A K A Q E A A A Q A A Y	9,000	-280
i+4 K,E	A A Q A A K A Q A E A A Q A A Y	10,350	-400
i+5 K,E	A A Q A A K A Q A A E A Q E A Y	6,900	
i+3 D,K	A A Q A A D A Q K A A A Q A A Y	8,200	-120
i+4 D,K	A A Q A A D A Q A K A A Q A A Y	9,200	-240
i+5 D,K	A A Q A A D A Q A A K A Q A A Y	7,400	
i+3 K,D	A A Q A A K A Q D A A A Q A A Y	8,400	-400
i+4 K,D	A A Q A A K A Q A D A A Q A A Y	10,300	-580
i+5 K,D	A A Q A A K A Q A A D A Q E A Y	5,800	
i+3 E,H	A A Q A A E A Q H A A A Q A A Y	4,500	-300
i+4 E,H	A A Q A A E A Q A H A A Q A A Y	4,100	-190
i+5 E,H	A A Q A A E A Q A A H A Q A A Y	3,600	
i+3 H,E	A A Q A A H A Q E A A A Q A A Y	4,400	-500
i+4 H,E	A A Q A A H A Q A E A A Q A A Y	5,300	-650
i+5 H,E	A A Q A A H A Q A A E A Q E A Y	2,300	

helix content
(0°C, 0.01 M NaCl,
pH 5.5/7.0)

Interactions
(0.01 M NaCl)

Waggawagga scoring

Stabilization / destabilization by amino acid interactions

Source residue	Target residue	Interaction	Score	Stabilization
H/K/R	E	i+4	1.0	strong
E	K/R	i+4	1.0	strong
H/K/R	E	i+3	0.75	medium
E	K/R	i+3	0.75	medium
K/R	D	i+4	0.75	medium
K/R	D	i+3	0.5	weak
D	K/R	i+4	0.75	medium
D	K/R	i+3	0.5	weak
E	H	i+3	0.5	weak
Q	E	i+4; i+3	0.5	weak
E	Q	i+4; i+3	0.5	weak
network stabilization (oppositely charged residues)		i, i+3, i+6	1.5	network
		i, i+3, i+7	0.25	network
		i, i+4, i+7	0.25	network
		i, i+4, i+8	2.0	network
hydrophobic seam		i, i+3, i+6	-0.5	destabilizing
		i, i+3, i+7	-0.5	destabilizing
		i, i+4, i+7	-0.5	destabilizing
		i, i+4, i+8	-0.5	destabilizing

Stabilization / destabilization by helix-forming propensity

Residue	Score	Stabilization
A	0.25	stabilizing
G	-0.5	destabilizing
P	-0.75	destabilizing
